# Pneumatosis Intestinalis: Not Always a Surgical Indication

**DOI:** 10.1155/2012/719713

**Published:** 2012-11-12

**Authors:** Haijing Zhang, Stephanie L. Jun, Todd V. Brennan

**Affiliations:** ^1^Department of Surgery, Duke University Medical Center, Durham, NC 27710, USA; ^2^Department of Radiology, University of California San Francisco, San Francisco, CA 94122, USA

## Abstract

We present a case of pneumatosis intestinalis (PI) of the colon in the setting of inflammatory bowel disease that was treated with medical management rather than emergent surgery. While the reflex response to extraluminal air in the abdomen is abdominal exploration, consideration of the clinical context in which PI is discovered and an understanding of a complete differential diagnosis of the sources of PI is critical to avoiding unnecessary surgery.

## 1. Introduction

Pneumatosis intestinalis (PI), also referred to as pneumatosis cystoides intestinalis, pneumatosis coli, and intestinal emphysema, is defined as the presence of extraluminal bowel gas that is confined within the bowel wall. The small intestine (42%) is most commonly involved followed by colon (36%), with involvement of both in 22% [1]. PI is an alarming radiological finding that usually prompts an emergent surgical consultation for concerns of bowel ischemia and impending bowel rupture. However, there is a wide spectrum of causes of PI ranging from the benign to the life-threatening. PI may be caused by bowel ischemia, mechanical trauma, inflammatory/autoimmune bowel disease, intestinal neoplasms, bowel infection, obstructive pulmonary disease, or drug-induced, including immunosuppression, therapy [1, 2]. Differentiating these causes is critical in directing an appropriate care plan. Complications are present in 3% of PI patients and include pneumoperitoneum, bowel obstruction, volvulus, intussusception, and hemorrhage [3]. Due to the risk of these emergent complications, suspected PI patients should be carefully evaluated for possible surgery. In a prospective review of patients with PI, bowel necrosis requiring surgery was predicted by 5 findings: an acute abdomen per history and physical, metabolic acidosis (pH < 7.3,  HCO_3_ < 20), elevated lactate, elevated serum amylase, and presence of portal venous gas [4]. For symptomatic PI of mild-to-moderate severity, treatment of the underlying disease with administration of antibiotics, oxygen therapy, and elemental diet may be sufficient for PI resolution. Here we describe an elderly patient with benign PI in the setting of inflammatory bowel disease.

## 2. Case Presentation

**Figure 1:**
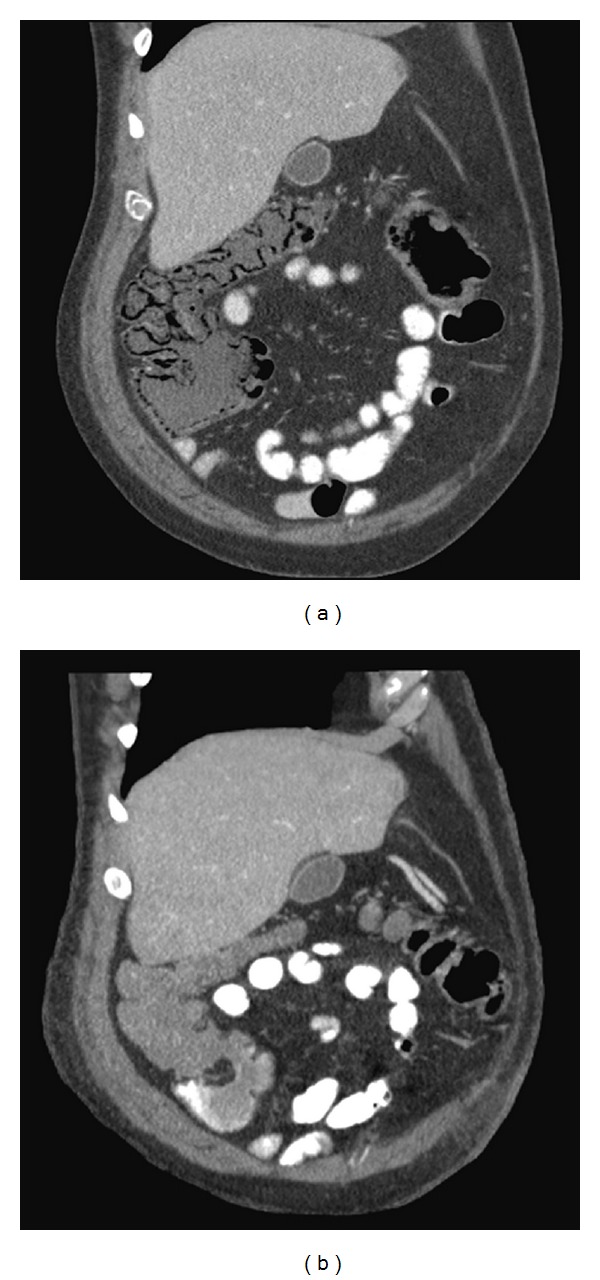
(a) CT scan of the abdomen 2 days following high-dose intravenous steroids shows extensive PI of right colon. Intramural gas tracking is visualized parallel to the bowel mucosa in the ascending and transverse colon. (b) Repeat CT scan after 3 days bowel rest and tapering of steroids and shows resolution of PI. No evidence of intramural gas along the colonic mucosa is seen.

A 60-year-old man was admitted for a flare of Crohn's disease, with pancolitis confirmed by colonoscopy with biopsy. He was discharged on oral prednisone, but was readmitted one week later for persistent abdominal pain, diarrhea, and a low-grade fever (38.1°C). A computed tomography (CT) scan of the abdomen on readmission showed thickening of the transverse, descending, and sigmoid colon. The right colon was normal at the time. After starting high-dose intravenous steroids (hydrocortisone 100 mg every 8 hours) and intravenous antibiotics (Cefazolin and Metronidazole), the patient defervesced and his symptoms improved. Two days later, a repeat CT performed for an elevated white blood cell count (18,000/uL) revealed extensive PI of the right colon (Figure 1(a)). Because his symptoms and physical exam findings were improving, the patient was treated with bowel rest and intravenous antibiotics while the steroids were tapered. After three days of close observation, a repeat CT scan demonstrated complete resolution of the PI (Figure 1(b)). The patient's symptoms resolved with medical management and he was discharged on maintenance oral prednisone.

## 3. Discussion

In PI, extraluminal gas predominantly localizes to the submucosal and subserosal planes of the small or large intestine, but can also localize to the muscularis propria [5]. While the pathophysiology of PI has been debated, it appears to be related to the breakdown of the mucosal and immunological barrier of the intestines, especially in the setting of increased intraluminal pressure. In our patient, the autoimmune inflammatory process of Crohn's disease and immunosuppression with high-dose steroid therapy likely contributed to PI etiology. Although the causality of high-dose corticosteroids in PI is not yet established, postulated mechanisms suggest that immunosuppression of antimicrobial defenses lead to intramural infection [6] and impairment of the intestinal wall barrier [7]. Our patient's prior colonoscopy with biopsy can be a contributing factor to developing PI as well, as recent biopsy increases risk of gas dissection into submucosa by compromising colonic mucosal integrity [8]. Based on a PubMed search, only 4 case reports [9–12] in English journals of PI associated with Crohn's disease have been presented in the past 10 years, with supportive therapy and nonsurgical resolution achieved in at least 3 of 4 cases [10–12].

Most cases of PI are asymptomatic [13] such that the diagnosis of PI may be an incidental radiographic or endoscopic finding. Patients with PI may also present only with symptoms of the underlying disease. The location of PI within the gastrointestinal tract can dictate the associated symptoms. Patients with small intestine PI most frequently present with vomiting (60%), abdominal distension (59%), weight loss (55%), and abdominal discomfort (53%). Patients with colonic PI most commonly present with symptoms of diarrhea (56%), hematochezia (50%), abdominal discomfort (32%), and abdominal distension (28%) [14]. While multiple imaging modalities are capable of detecting PI, CT scan with or without intravenous contrast is more sensitive than plain film, MRI, or ultrasound in diagnosing and characterizing the extent of PI [15]. The radiological characteristics of PI on CT are intramural gas tracking parallel to the bowel mucosa as shown (Figure 1(a)). A review of 44 pediatric PI cases showed that additional CT features may distinguish between emergent and mild PI; these CT findings include thickening of bowel wall, free peritoneal fluid, extent of PI, and soft tissue stranding of peri-intestinal tissues [16]. Though not seen in this patient, gas in the portal and mesenteric veins is a poor prognostic factor [17], and is more frequently associated with ischemic bowel, especially in pediatric patients in the setting of acute necrotizing enterocolitis [4]. Signs of bowel ischemia, bowel necrosis, and bowel perforation resulting in intraperitoneal air indicate an emergent setting in which surgery may be appropriate. Other complications of PI such as bowel obstruction may indicate surgical intervention or endoscopic puncture and sclerotherapy of the cysts [18]. However, as in this case, PI patients with cardiovascular stability and an unimpressive abdominal exam may be closely observed and treated with bowel rest and antibiotics.

## 4. Conclusion

Overall, PI is a rare radiological finding and one that occurs in wide spectrum of clinical disorders. In the setting of the acute abdomen with coexisting systemic sepsis, necrotic bowel must be suspected and emergent operative management pursued. However, in the setting of a nonacute abdomen and a stable patient, benign causes of PI need to be considered in the differential diagnosis. As illustrated by the presented case, the radiological finding of PI is not always an indication for surgery and may be treated with medical therapy alone in many clinical circumstances.

## References

[1] Braumann C, Menenakos C, Jacobi CA (2005). Pneumatosis intestinalis—a pitfall for surgeons?. *Scandinavian Journal of Surgery*.

[2] Ho LM, Paulson EK, Thompson WM (2007). Pneumatosis intestinal is in the adult: benign to life-threatening causes. *American Journal of Roentgenology*.

[3] Galandiuk S, Fazio VW (1986). Pneumatosis cystoides intestinalis: a review of the literature. *Diseases of the Colon and Rectum*.

[4] Knechtle SJ, Davidoff AM, Rice RP (1990). Pneumatosis intestinalis. Surgical management and clinical outcome. *Annals of Surgery*.

[5] Koreishi A, Lauwers GY, Misdraji J (2007). Pneumatosis intestinalis: a challenging biopsy diagnosis. *American Journal of Surgical Pathology*.

[6] John A, Dickey K, Fenwick J, Sussman B, Beeken W (1992). Pneumatosis intestinalis in patients with Crohn's disease. *Digestive Diseases and Sciences*.

[7] Shimojima Y, Ishii W, Matsuda M, Tojo K, Watanabe R, Ikeda SI (2011). Pneumatosis cystoides intestinalis in neuropsychiatric systemic lupus erythematosus with diabetes mellitus: case report and literature review. *Modern Rheumatology*.

[8] Meyers MA, Ghahremani GG, Clements JL, Goodman K (1977). Pneumatosis intestinalis. *Gastrointestinal Radiology*.

[9] Arena V, Pennaccia I, Abenavoli L (2011). ... And suddenly a tree!. *International Journal of Surgical Pathology*.

[10] Breitinger A, Kozarek R, Hauptman E (2003). Pneumatosis cystoides intestinalis in Crohn's disease. *Gastrointestinal Endoscopy*.

[11] Hwang J, Reddy VS, Sharp KW (2003). Pneumatosis cystoides intestinalis with free intraperitoneal air: a case report. *American Surgeon*.

[12] Gelfond D, Blanchard SS, Malkani A (2011). Pneumatosis intestinalis: a rare presentation of Crohn disease exacerbation. *Journal of Pediatric Gastroenterology and Nutrition*.

[13] Heng Y, Schuffler MD, Haggitt RC, Rohrmann CA (1995). Pneumatosis intestinalis: a review. *American Journal of Gastroenterology*.

[14] Jamart J (1979). Pneumatosis cystoides intestinalis. A statistical study of 919 cases. *Acta Hepato-Gastroenterologica*.

[15] Pear BL (1998). Pneumatosis intestinalis: a review. *Radiology*.

[16] Olson DE, Kim YW, Ying J, Donnelly LF (2009). CT predictors for differentiating benign and clinically worrisome pneumatosis intestinalis in children beyond the neonatal period. *Radiology*.

[17] Nelson SW (1972). Extraluminal gas collections due to diseases of the gastrointestinal tract. *The American Journal of Roentgenology*.

[18] Johansson K, Lindstrom E (1991). Treatment of obstructive pneumatosis coli with endoscopic sclerotherapy: report of a case. *Diseases of the Colon and Rectum*.

